# Left Main Coronary Artery Percutaneous Coronary Intervention

**DOI:** 10.3390/jcm11216584

**Published:** 2022-11-07

**Authors:** Arnaud Ferrante, Paul Guedeney

**Affiliations:** Sorbonne Université, ACTION Group, INSERM UMRS 1166, Hôpital Pitié-Salpêtrière (AP-HP), Institut de Cardiologie, 75013 Paris, France

Left main coronary artery (LMCA) revascularization remains a critical part of coronary artery disease (CAD) management as it improves patients’ prognoses by reducing all-cause and cardiac mortality [1]. While it has long remained the prerogative of the surgeon, the evolution of percutaneous coronary intervention (PCI) techniques and the improvement of both stent technology and antithrombotic treatment have led to a debate on the roles that PCI and coronary artery bypass graft (CABG) should play in the treatment of LMCA lesions [2]. Recently, several randomized controlled trials (RCT) and subsequent patient- and study-level meta-analyses have demonstrated an equipoise between PCI and CABG in a selected, low-risk subgroup of patients in terms of all-cause and cardiovascular mortality, major adverse cardiac events, myocardial infarction and stroke, albeit with a higher rate of subsequent revascularization with PCI [3,4]. Deciding between PCI and CABG is essentially based on the patients’ comorbidities, particularly diabetes mellitus, the surgical risk as assessed by the STS score or the EuroSCORE II, left ventricular ejection fraction (LVEF), the anatomical complexity assessed by the SYNTAX score and the need for concomitant valvular or aortic surgery [4]. Bifurcation lesions are frequent with LMCA and have been associated with a higher risk of target lesion failure (TLF) compared to other non-LMCA bifurcation lesions, thus emphasizing the crucial role of the adequate use of the currently available armamentarium for PCI to improve outcomes [5,6].

One of the first aspects is to adequately evaluate LMCA by using intravascular imaging with intravascular ultrasound or optical coherence tomography, which may be the only option in case of ostial lesion. Prior to PCI, intravascular imaging may provide useful information on lesion characteristics such as plaque extent and severity, minimal lumen area, cross-sectional characteristics and the involvement of the side branches. All these information may help define the optimal PCI strategy by determining the diameter and length of the stents and detailing their landing zones. After PCI, intravascular imaging may still identify suboptimal results with incomplete stent deployment, malposition, edge dissection, thrombus or a strut protrusion [7]. Another way to evaluate lesions of the LMCA is to assess their functional significance with the use of fractional flow reserve or instantaneous wave-free ratio, with cut-off values of, respectively, ≤0.80 and ≤0.89 [8]. Physiological assessment is also useful after the PCI to ensure a good hemodynamic result on the treated lesion or a bifurcation branch. Other techniques such as the measurement of the anterior wall thickness of the LMCA by transthoracic echocardiography have also been described, with an adequate sensibility for the diagnosis of fibro-calcific plaque, although further validation is necessary [9].

Because of its specific anatomical characteristics, lesions of the LMCA distinguish from others especially with a greater volume of atherosclerotic plaque, more frequent and severe calcifications, and a common involvement of the distal bifurcation. The latter raises the issue of a one or two stents for the PCI strategy and although some observational studies have reported improved outcomes with a single-stent strategy [5], a dedicated randomized trial demonstrated a significant reduction in TLF and stent thrombosis with a two-stent strategy with the use of the DK-crush technique [10]. Considering the large plaque burden and the degree of calcification, plaque modification strategies before stenting, such as rotational, orbital, laser atherectomy or lithotripsy, may be paramount in the setting of LMCA PCI to ensure a proper debulking and good stent expansion, which has been associated with better outcomes [11].

Much remains to be done to improve outcomes in the setting of LMCA. Although CABG may remain the gold standard in cases of complex lesions and/or patients with diabetes mellitus, PCI remains a valid option for patients too frail to undergo surgery or presenting low-risk lesion. This series of ten articles may shed some light on the current issues and novel therapeutic strategies that may be used for PCI of the LMCA.
